# The PGPR *Stenotrophomonas maltophilia* SBP-9 Augments Resistance against Biotic and Abiotic Stress in Wheat Plants

**DOI:** 10.3389/fmicb.2017.01945

**Published:** 2017-10-09

**Authors:** Rajnish P. Singh, Prabhat N. Jha

**Affiliations:** Department of Biological Science, Birla Institute of Technology and Science, Pilani, Pilani, India

**Keywords:** PGPR, ACC deaminase, ERIC-PCR, induced systemic tolerance, salt stress, osmolytes

## Abstract

Certain plant growth promoting bacteria have ability to ameliorate abiotic and/or biotic stressors, which can be exploited to enhance plant growth and productivity of the plants under stress conditions. Therefore, the present study aimed to examine the role of a rhizospheric bacterial isolate SBP-9 isolated from *Sorghum bicolor* (i) in promoting the wheat plant growth under salinity stress, and (ii) in enhancing the defense response in wheat against fungal pathogen “*Fusarium graminearum*.” The test isolate possessed plant growth promoting (PGP) traits including ACC deaminase (ACCD), gibberellic acid, indole acetic acid (IAA), siderophore, and inorganic phosphate solubilization. Under salt (NaCl) stress, inoculation of this isolate to wheat plant significantly increased plant growth in terms of various growth parameters such as shoot length/root length (20–39%), fresh weight/dry weight (28–42%), and chlorophyll content (24–56%) following inoculation of test isolate SBP-9. Bacterial inoculation decreased the level of proline, and malondialdehyde, whereas elevated the antioxidative enzymatic activities of superoxide-dismutase (SOD; 28–41%), catalase (CAT; 24–56%), and peroxidase (POX; 26–44%). Furthermore, it also significantly decreased the Na^+^ accumulation in both shoot and roots in the range of 25–32%, and increased the K^+^ uptake by 20–28%, thereby favoring the K^+^/Na^+^ ratio. On the other hand, the test isolate also enhanced the level of defense enzymes like β-1, 3 glucanase, phenylalanine ammonia lyase (PAL), peroxidae (PO), and polyphenol oxidase (PPO), which can protect plants from the infection of pathogens. The result of colonization test showed an ability of the test isolate to successfully colonize the wheat plants. These results indicate that *Stenotrophomonas maltophilia* SBP-9 has potential to promote the wheat growth under biotic and abiotic (salt) stressors directly or indirectly and can be further tested at field level for exploitation as bioinoculant.

## Introduction

Soil salinity is a naturally occurring problem in arid and semiarid regions and continuously increasing due to the exhaustive use of chemical fertilizers, and improper irrigation management (Bharti et al., 2013). It has reduced the world's irrigated area by 1–2% every year, thereby severely affecting the agriculture production (FAO, 2005). According to the UN (United Nations), at least 50% of the world's arable lands are subjected to salinity stress (Flowers and Yeo, 1995). The amount of land affected by salt represents almost 20% of the world's cultivated area and it is increasing as a direct attribute of irrigation and agricultural malpractices (Glick, 2014). The plants growing under salinity and other stresses (both biotic and abiotic) suffer from a high level of ethylene termed as “stress ethylene” (Jackson, 1985) which adversely affects the growth and various metabolic processes leading to senescence in plants. Enhanced level of ethylene is one of the important markers for both abiotic and biotic stressors (Sheehy et al., 1991; Heidstra et al., 1997; Ma et al., 2003).

Certain PGPR (plant growth promoting rhizobacteria) strains equipped with enzyme ACCD can degrade the ACC to ammonia and α-ketobutyrate, and thereby minimize the level of stress ethylene (Mayak et al., 2004; Barnawal et al., 2012). Therefore, these PGPR reduce the inhibitory effect of stress ethylene generated under adverse environmental conditions, and reduce the endogenous level of ACC in plants. The effectiveness of bacterial isolate in plant growth stimulation and the alleviation of salt stress in salt-rich soils have been demonstrated in previous studies (Egamberdieva, 2008; Nadeem et al., 2010; Barnawal et al., 2014).

In addition to stress ethylene, increasing soil salinity affects the multitude of responses in plants including the several biochemical and physiological processes like synthesis of protein, lipid metabolism, photosynthesis, and ionic homeostasis (Parida and Das, 2005). It also restricts the water uptake and induces the toxicity of Na^+^ (Ashraf et al., 2004; Mayak et al., 2004). PGPR can overcome the harmful effects of salinity by maintaining a favorable ratio of K^+^/Na^+^ ions amenable for plant growth under high salt levels (Mayak et al., 2004), accumulation of compatible solutes or osmolytes, stabilizing membrane lipids (Hincha et al., 2003; Bano and Fatima, 2009), maintenance of redox potential (Colmer et al., 1995; Yancey, 2005), free radicals scavenging (Smirnoff and Cumbes, 1989), binding to toxic metals (Geddie and Sutherland, 1993; Sharma and Dietz, 2006; Karthikeyan et al., 2007), and induction of transcription factors under stress responses (Gupta et al., 2012).

Among PGPR, plant growth promoting and biocontrol potential of *Stenotrophomonas* sp. has been reported in earlier studies which demonstrated that it can be used as an effective bioinoculants for plant growth promotion and controlling the wide range of plant pathogenic fungi and, therefore have great potential for biotechnology applications (Ryan et al., 2009; Berg et al., 2010). Plant growth promotion ability of *Stenotrophomonas rhizophila* strain DSM14405^T^ was observed in the high salt rich soils of Uzbekistan at levels up to 180% (Egamberdieva et al., 2011). Similarly, Singh et al. (2013) showed the Quorum quenching (QQ) activity against *Chromobacterium violaceum* CV026 and anti-biofilm activities of a rhizobacterium *Stenotrophomonas maltophilia* BJ01. Brooke et al. (2017) also encountered the multifarious approach of bacterium *S. maltophilia*. A previous report (Alavi et al., 2013) suggested that *S. rhizophila* possesses certain genes responsible for beneficial plant-microbe interaction, transport of osmoprotectants, biocontrol activity, and colonization. However, the detail characterization and mechanism of plant growth stimulated by *Stenotrophomonas* sp. under salinity stress conditions is still lacking.

Besides, rhizobial inoculants have also been reported to suppress disease by eliciting the induced systemic resistance (ISR) against a number of plant diseases (Kumari and Srivastava, 1999). The induction and increased production of defense-related enzymes during ISR are known to play a crucial role in host resistance (Chen et al., 2000; Ramamoorthy et al., 2002). Bacteria belonging to genera like *Pseudomonas* and *Bacillus* sp. have been known to induce resistance to bacterial and fungal pathogens. However, *S. maltophilia*-mediated elicitation of ISR in wheat is still unknown. A recent study has shown the biocontrol behavior of *S. maltophilia* (PD4560) against *Ralstonia solanacearum* by proteolytic enzyme production and through induction of pathogenesis related (PR) genes (Elhalag et al., 2016). The role of PR proteins in helping plants to counteract the stressed condition has been addressed in another study (Koike et al., 2002). However, the detailed characterization and its priming effect against fungal pathogen are at primary level.

The presence of salts in the soil is a major problem for agricultural crops like wheat that leads to a major drop in wheat grain yield in the range of 20–43%, with an overall average loss of 40%. The production of wheat in India is 80.2 million ton annually, which is about 12% of total world production (http://dacnet.nic.in). Therefore, there is an urgent need for an effective bioinoculant for its/their ability to promote wheat plant growth under saline stress. We hypothesized that halotolerant bacteria are able to ameliorate salinity stress and therefore, can be used as an effective tool for development of bio-formulations that can be tested in field trials. In this study, we made an attempt to explore the potential of a halotolerant ACC deaminase producing strain of *S. maltophila* for its multifarious PGP (plant growth promoting) traits to promote wheat plant growth under saline stress. For this, we examined the protective role of *S. maltophila* using diverse physiological and biochemical mechanisms, and to evaluate its efficacy to confer abiotic stress tolerance, particularly in wheat plant. In addition, the strain used in the present study was shown to induce ISR against fungal challenged wheat plants.

## Materials and methods

### Isolation of bacteria

The bacterial strain was isolated from the rhizospheric soil of *Sorghum bicolor*, commonly growing in the arid region of Rajasthan, India. ~1 g of soil sample was serially diluted (up to 10^−9^) with sterile distilled water and 100 μl of the suspension was spread on the LB-agar medium. The plates were incubated for 48–72 h at 30°C. A total of 15 bacterial colonies with varying morphologies were selected and further cultured in minimal medium (DF) containing 3 mM ACC (Sigma-Aldrich, USA) (Dworkin and Foster, 1958). ACC utilizing bacterial isolates were screened for the ACC deaminase assay and other plant growth properties. Based on ability to utilize ACC as a nitrogen source, ACCD activity and other PGP features, isolate SBP-9 was selected for detailed study.

### Biochemical characterization and identification of strain SBP-9

The test isolate was characterized by various biochemical tests (like methyl-red, Voges-Proskauer, Indole, citrate utilization, nitrate reductase, urease, oxidase, catalase, and gram staining using standard protocol (Harley and Prescott, 2002). Test of motility was also checked using standard procedure (Connelly et al., 2004). Test of carbohydrate utilization was performed using KB-009, carbohydrate utilization kit (Himedia, India). Antibiotic sensitivity test of isolate to standard antibiotics was evaluated using HTM-002, antibiotic sensitivity kit (Himedia, India). Antagonistic activity against certain fungal pathogens namely *Aspergillus flavus, Fusarium oxysporum, Fusarium moniliforme, Candida albicans, Penicillium citrium*, and *Fusarium graminearum* was determined by agar well-diffusion method. Molecular technology employing 16S rRNA amplification for identification of selected test organism was performed via polymerase chain reaction (PCR), following the standardized protocol (Singh et al., 2015). Taxonomic affiliation of SBP-9 was assigned (http://rdp.cme.msu.edu/seqmatch/seqmatch_intro.jsp) and the phylogenetic relationship was established (Tamura et al., 2013).

### Stress tolerance studies

The tolerance of the selected isolate toward various abiotic stressors like pH, temperature, and salinity was studied. Salt tolerance (1, 2, 4, 6, and 8% NaCl, w/v) was tested on DF-agar medium supplemented with ACC (3 mM). The strain was streaked on the solid-agar medium and visualized for the growth following incubation at 30°C for 2–3 days. Tolerance to varying temperatures was studied by streaking the isolate on tryptic-soy agar plates and incubated at different temperatures viz. 20–60°C. Tolerance to salt, pH, and temperature stress was also done by inoculating the strain in to tryptic soya broth medium and incubating at desired time interval. For pH studies, 100 μl of overnight grown culture (10^7^ CFU ml^−1^) was added to tryptic soya broth and pH of various ranges (5.0–10.0) was maintained by 2 N NaOH and 1 N HCl using the pH meter (Eutech, pH 1100). After 72 h, culture pellet was suspended in 2 ml of sterile water, and optical density (OD) was determined at 600 nm in a UV-Visible spectrometer (Jasco Corporation, Japan) to test the pH tolerance. Each culture was inoculated in triplicate sets.

### Test for plant growth promoting features

ACC deaminase activity of isolate SBP-9 was tested by measuring the amount of α-ketobutyrate production, a cleavage product of ACC (Honma and Shimomura, 1978), and protein concentration were determined using the Bradford method (Bradford, 1976). The ACC deaminase activity was expressed in terms of nmol of α-ketobutyrate mg^−1^ protein. Test of phosphate solubilization was performed in NBRIP (National Botanical Research Institute's Phosphate) medium supplemented with insoluble tricalcium phosphate and quantified as per the standard protocol (Mehta and Nautiyal, 2001). A standard curve was prepared using various concentrations of K_2_HPO_4_ (Merck, India). Test of IAA production was done by using Salkowsky's reagent (Gordon and Weber, 1951), and optical density of the resulting solution was measured spectrophotometrically at 530 nm using a Jasco-630 UV-visible spectrophotometer. A standard curve of IAA was used for measuring the IAA concentration in test samples using un-inoculated medium as a control. Gibberellic acid production was tested by the spectrophotometric method (Holbrook et al., 1961). Test for siderophore production was evaluated on chrome azurole S-agar (CAS-agar) plates and observed for formation of color zone around the point inoculated colony (Schwyn and Neilands, 1987). Assay for ammonia production was tested using Nessler's reagent (Cappuccino and Sherman, 1992). A preliminary test for nitrogen fixation ability of SBP-9 was done by growing on JNFb^−^ agar medium (Dobereiner, 1997). In addition, *nif* H gene was amplified using specific primers: Pol F (5′-TGCGAYCCSAARGCBGACTC-3′) and Pol R (5′-ATSGCCATCATYTCRCCGGA-3′) (Sigma–Aldrich), where Y = C/T, S = G/C, R = A/G, B = G/T/C.

### Physiological test of ACCD activity

The ACCD activity of SBP-9 was tested under various physiological conditions namely varying salt concentration, temperature, pH, and different incubation periods. To evaluate the activity under various salinity levels, different concentration of NaCl (2–8%) was supplemented in minimal medium containing 3 mM ACC, while for temperature assay SBP-9 was grown at different temperatures (25–45°C) in an incubator. Similarly, pH of the culture medium was adjusted with 2 N HCl and 1 M NaOH to attain pH 5.0 to 11.0. In addition, the enzymatic activity was also assessed under different incubation periods.

## Evaluation of plant growth promoting test

### Inoculum preparation and seed treatment

Effect of the bacterial isolate SBP-9 on the growth of wheat plant (*Triticum aestivum* L.) under salinity stress was tested in a controlled environment of plant growth chamber. Soil used for pot study was analyzed for its various physicochemical properties using Atomic Absorption Spectrophotometer (AAS). The soil was autoclaved at 121°C for 1 h for 3 consecutive days to kill any microbial presence. Sterility of the soil was checked by standard serial dilution method. Physico-chemical characteristics of soil used in pot were as follows: pH 7.20 ± 0.05, EC 0.161 ± 0.03 ds m^−1^, Olsen P 32.9 ± 1.7 mg kg^−1^, Total N 57 ± 2.0 mg kg^−1^, Total K 118.0 ± 3.1 mg kg^−1^, Zn 0.221 ± 0.003 mg kg^−1^, Cu 0.118 ± 0.003 mg kg^−1^, Fe 2.88 ± 0.04 mg kg^−1^, and Mn 0.916 ± 0.05 mg kg^−1^. Preparation of bacterial inoculum (OD 0.15) and seed treatment was performed according to Penrose and Glick (2003). Briefly, wheat (*T. aestivum* L.) seeds were surface sterilized by treating with 70% ethanol followed by 2% sodium hypochlorite (NaOCl) solution for 3 min. The sterilized seeds were thoroughly washed using sterile water to remove all traces of sodium hypochlorite. The surface-sterilized seeds of wheat were kept in the bacterial suspension for 1 h. Surface sterilized seeds treated with 0.03 M MgSO_4_ instead of bacterial suspension served as control. Twenty bacterized seeds were sown in each plastic pot (22 cm in height, 16 cm in diameter) filled with sterilized soil (400 g) and grown with 16:8 photoperiods for 15 days after seed germination at 24 ± 2°C. For striking the salt stress of 150 mM (T-1) and 200 mM (T-2), NaCl was added in Hoagland medium to achieve the desired concentration for providing the nutrient as well as imposing the salt treatment to experimental plants. A set of control plants with 0 mM NaCl (T-0) was also taken for comparative analysis. Pots were arranged in completely randomized block design with three replications in each treatment.

For measuring growth (root/shoot length) and biomass (fresh/dry weight), five randomly selected plants from each replicate were used. To estimate the chlorophyll content, fresh leaf samples of 500 mg (0.5 g) were ground thoroughly with 80% acetone and centrifuged at 9,000 g for 10 min at 4°C. The absorbance of collected supernatant were read at 645 and 663 nm using a UV-Visible spectrometer (Jasco Corporation, Japan) to estimate total chlorophyll content (Moran and Porath, 1980). The same was calculated as follows:

$$\begin{array}{l} \text{Chlorophyll}=\left[8.02\times {\text{A}}_{633}\right]-\left[20.02\times {\text{A}}_{645}\right] \end{array}$$

### Ionic accumulation analysis

To conduct an ionic analysis of plants treated with salt stress, roots were washed twice for eight to ten min in ice-cold 20 mM CaCl_2_ to allow the exchange of cell wall bound K^+^ and Na^+^, and finally washed five to six times with autoclaved Milli-Q water. Roots and shoots were separated and oven dried at 70°C for 48 h. Afterward, 1 g plant tissue was ground in liquid N_2_ and digested in a mixture of 30% H_2_O_2_, 65% HNO_3_, and de-ionized water in a ratio of 1:1:1 at 120°C for 2 h to a final volume of 12 ml in a microwave digester. Ions namely Na^+^, and K^+^ were estimated by AAS (AAS 2380, Perkin Elmer, USA) at NHRDF (National Horticultural Research and Development Foundation; Nashik, India).

### Antioxidant assay

Plant leaves (0.5 g) were extracted in the buffer containing 5 ml of 50 mM phosphate buffer (pH 7.0) supplemented with 1% polyvinylpyrrolidone (PVPP). The crude extract was centrifuged at 10,000 g for 15 min at 4°C, and the obtained supernatant was used for the antioxidant assay. Superoxide dismutase assay, which is based on its ability to inhibit the photochemical reduction of nitro blue tetrazolium (NBT), was carried out as per the method of Beauchamp and Fridovich (1971) with minor modifications. The reaction mixture containing 100 μl of enzyme extract in 50 mM phosphate buffer (pH 7.8), 13 mM methionine, 75 μM NBT, 2 μM riboflavin, and 0.1 mM EDTA was made up to 3 ml. The assay mixture was incubated at room temperature under two fluorescent tubes (15 W) for 10 min to allow the development of purple color formazan which was then measured at 560 nm against the blank. One hundred microliters of distilled water was used as blank instead of enzyme extract. The reaction was stopped by switching off the light. The activity was measured in terms of inhibition of 50% of NBT photo-reduction at 560 nm and expressed as units per mg of protein.

Catalase test was determined by monitoring the reduction in the absorbance of H_2_O_2_ at 240 nm wavelength. The reaction mixture (3 ml) consisted of 100 μl enzyme extract with 50 mM phosphate buffer (pH 7.8), 0.1 mM EDTA and 12.5 mM H_2_O_2._ The activity was calculated based on an extinction coefficient of 0.04 mM^−1^ at 240 nm. The peroxidase (POD) activity in the extract was determined by the method of Kar and Mishra (1976) with minor modifications. The assay mixture consisted of 100 μl of enzyme extract with 0.1 M phosphate buffer, 0.1 mM pyrogallol, 5 mM H_2_O_2_ and incubated for 5 min at 25°C. For turning off the reaction 1.0 ml of 2.5 N H_2_SO_4_ was used and observed indigo color formed was read at 420 nm against blank containing water in place of enzyme extract.

### Biochemical analysis of plant

Proline content in the leaves was determined following the standard protocol (Bates et al., 1973) with minor modifications. A 0.5 g of fresh leaves were homogenized in 3 ml of 5% (w/v) sulfosalicylic acid and centrifuged at 8,500 g for 10 min. Five hundred microliters of resulting supernatant was made up to 1 ml with sterile water and gently vortexed with 2 volumes of 2% ninhydrin. The mixture was boiled for 30 min at 100°C. After cooling, an equal volume of toluene was added to the mixture and upper aqueous phase was used for taking absorbance at 520 nm in a spectrophotometer (Jasco Corporation, Japan). The proline content was estimated by comparing with a standard curve of L-proline (Sigma-Aldrich, USA) as standard.

The extent of lipid peroxidation was calculated by measuring the malondialdehyde (MDA) content formed through thiobarbituric acid reaction following method of Hodges et al. (1999) with minor modification. The alcoholic extract (1 ml) of leaves was mixed with 1 ml of 0.5% thiobarbituric acid containing 20% trichloroacetic acid and heated up to 90°C for 30 min. Following cooling, the sample was centrifuged at 5,000 g for 5 min and the supernatant was read at 400, 532, and 600 nm. The MDA concentration was determined by its molar extinction coefficient (155 mM^−1^ cm^−1^) and expressed as mmol MDA g^−1^ fresh weight (FW).

## Defense assay

### Inoculum preparation and plantlet treatment

The wheat seeds were sterilized as per above mentioned protocol and left for germination in dark for about 4–5 days in a moist condition. The germinated seedlings were grown 9 days in controlled conditions with 16:8 photoperiod at 24 ± 2°C. Preparation of bacterial inocula was done as per above section. For fungus treatment, *F. graminearum* was grown in potato dextrose medium at 28°C for 3–4 days. After the growth, the culture was harvested at 7,000 g for 15 min. The obtained pellet was washed with sterile 1X phosphate buffer saline (PBS) and re-suspended in the buffer to attain 4,000 spores/ml of fungus. On the 9th day, germinated plants were challenged with the pathogen in bacterium primed plants and in control plants. A separate set of bacterium-inoculated and control plants were also taken for comparative analysis of defense enzymes during experimental study.

Plant samples were crushed in liquid nitrogen at every 24 h period for the next 6 days after inoculation. All the samples were assayed in triplicate sets. Each of the crushed plant material was aliquoted into four 1.5 ml eppendorf tubes (three for each PR protein assay) and stored at −70°C for later use. These tubes were then re-suspended in respective buffers with 0.5 g plant material being suspended in 1 ml buffer.

### β 1, 3-glucanase assay

0.5 g of crushed plant tissue was extracted in 50 mM sodium acetate buffer (pH 5.0), and centrifuged at 12,000 g for 15 min at 4°C. The extract of 80 μl was mixed with 40 μl of 4% laminarin and kept at 40°C for 10 min. For stopping the reaction, 300 μl of dinitrosalicylic acid reagent was added in the mixture and heated for 10 min. To stabilize the color, 40 μl of 40% sodium potassium tartarate was added and diluted three times with distilled water to take its absorbance at 575 nm (Kurt, 1991).

### Phenylalanine ammonia lyase assay (PAL)

The crushed plant tissue (0.5 g) was re-dissolved in 50 mM Tris buffer (pH 8.8). The homogenate was centrifuged at 12,000 g for 15 min at 4°C, and supernatant was used for analysis. The reaction mixture consisted of 176 μl of 70 mM Tris pH 8.8, 70 μl of 10 mM phenylalanine, and 100 μl of enzyme extract. The reaction was allowed to proceed at 30°C for 60 min after which it was stopped by adding 200 μl of 2 N HCl. Finally, the reaction mixture was extracted with 200 μl of toluene by vortexing for 15 s, and the mixture was centrifuged at 2,000 g for 5 min to separate the phases. The upper phase was used for estimating the amount of cinnamic acid at 290 nm (Ramamoorthy et al., 2002).

### Peroxidase assay (PO)

The extract of plant tissue (0.5 g) was made in 10 mM sodium phosphate buffer (pH 6.0), and homogenate was centrifuged at 12,000 g for 15 min at 4°C. The reaction mixture consisted of 0.25% guaiacol, 10 mM sodium phosphate buffer, and 0.1 M H_2_O_2_ in 2.9 ml to which 0.1 ml of enzyme extract was added (Hammerschmidt et al., 1982).

### Polyphenol oxidase assay (PPO)

0.5 g of crushed plant tissue was re-dissolved in 100 mM sodium phosphate buffer (pH 6.5), centrifuged at 12,000 g for 15 min at 4°C and supernatant was used for analysis. The reaction mixture consisted of 0.45 ml of 100 mM sodium phosphate buffer (pH 6.5), 50 μl of 0.01 M tert-butyl catechol, and 40 μl of enzyme extract (Mayer, 2006).

## Root colonization

Root colonization of inoculated bacterium was determined on the 15th day of plant growth using serial dilution plating technique on NA-agar medium and number of viable cells was estimated as colony forming units (CFU) as described (Somasegaran and Hoben, 1994). Additionally, for confirming the identity of the colonized bacterium, ERIC-PCR (enterobacterial repetitive intergenic consensus) of recovered bacterial colonies from treated plants was performed as per standardized protocol (Singh et al., 2015). The treated plants were up-rooted and gently washed in sterile Milli-Q water to remove the soil particles and loosely bound bacteria from the roots. The g-DNA of the bacterial treated plant was isolated by bacterial DNA isolation kit. Pure culture of test isolate was also used as positive control.

## Statistical analysis

The experiment was conducted in completely randomized designs, and results were expressed as means ± standard errors of three independent replicates. The difference between means in each treatment was analyzed by analysis of variance (ANOVA) and subsequently by Duncan's multiple range tests (*p* = 0.05, <0.05, <0.01) using by a DPS statistical software package (version 11.0).

## Results

### Isolation and primary characterization of bacteria

Based on the luxuriant growth on DF medium containing ACC, bacterial isolate SBP-9 was selected for further study. Continuous growth of SBP-9 on DF-ACC agar plate after several sub-culturing illustrated its ability to utilize ACC as a nitrogen source. It was found positive for the test of lipase, urease, and nitrate reductase, whereas negative for indole, methyl red, Voges-Proskauer, amylase, and catalase. In addition, SBP-9 also showed pectinolytic and cellulolytic (exoglucanase & endoglucanase) activities. It showed growth up to 50°C, while optimal temperature (based on OD) for the growth was 30°C. The isolate was able to tolerate salt concentration up to 8% NaCl, while the optimum growth was observed at 4% NaCl. Similarly, pH tolerance was found in a range of pH 6–11. Moreover, antibiotic sensitivity profiling of the isolate SBP-9 showed its resistance to kanamycin, ampicillin, tetracycline, gentamycin, whereas sensitivity to chloramphenicol, streptomycin, and erythromycin (Table 1). Among the tested carbon sources, SBP-9 utilized various carbon sources that have been summarized in Supplementary Table 1. The test isolate inhibited the growth of *F. oxysporum, F. graminearum*, and *P. citrium* and showed the swimming, swarming, and twitching motilities.

**Table 1:** Biochemical characterization of isolate SBP-9.

**Characteristic (s)**	**Activity**
Gram test	–
Indole	–
MR	–
VP	–
Amylase	–
Lipase	+
Urease	+
Catalase	–
Nitrate reductase	+
Max. temperature tolerance (°C)	50
Salt (NaCl) tolerance(%)	8
pH tolerance	6–11
**MOTILITY**	
Swimming	+
Swarming	+
Twiching	+
**ANTIBIOTIC RESISTANCE**	
Chloramphenicol	+
Streptomycin	+
Erythromycin	+
Tetracycline	++
Kanamycin	++
Gentamycin	++
Ampicillin	++

*+, sensitive; ++, resistant; +, positive; −, negative*.

### Identification and phylogenetic analysis

The PGPR isolate SBP-9 identified as *S. maltophilia* showed 100% identity with other reported gene sequences (16S rRNA) of *Stenotrophomonas* sp. (Supplementary Figure 1). Threshold of >98% sequence match with type strain was considered for identification. The sequence of resulting amplicon (580 bp) was submitted to the Genbank database under the accession number KJ950710.

### Plant growth promoting features

Quantitative value for ACC deaminase activity of isolate SBP-9 was determined as 362 ± 4.1 nmol of α-KB mg^−1^ protein h^−1^. Formation of a clear zone around the streaked colony on media supplemented with an insoluble form of phosphate (tri-calcium phosphate) indicated mineral phosphate solubilizing activity. On quantification of phosphate solubilization, it solubilized 10.73 ± 2.34 μg ml^−1^. Among the phytohormones, the isolate produced 3.16 ± 0.12 μg ml^−1^ indole-3-acetic acid, and 5.40 ± 1.10 μg ml^−1^ gibberellic acid. The appearance of orange-halo zone on the CAS-agar plate was considered as positive for siderophore production (Supplementary Figure 2). Continuous growth for several generations on N^−^ medium indicated an ability of the test isolate to fix atmospheric nitrogen. Moreover, amplification of the *nif*- H gene in *S. maltophilia* SBP-9 supports the nitrogen-fixing potential at the molecular level. The desired band of 300 bp corresponding to the *nif*-H gene was obtained by using universal primers for the *nif*-H gene (Supplementary Figure 3). In addition, it was also positive for ammonia, and HCN production (Table 2).

**Table 2:** Plant growth promoting traits of strain SBP-9.

**Plant growth promoting traits**	**Activity**
ACCD activity (nmol of α-KB mg^−1^ Pr·hr^−1^)	362 ± 4.1
IAA production (μg/ml)	3.16 ± 0.12
Gibberellic acid (μg/ml)	5.40 ± 1.10
Phosphate solubilization (μg/ml)	10.73 ± 2.34
Siderophore index	+
HCN production	+
Ammonia production	+

*±, standard deviation; +, positive*.

### Physiological enzyme activities

**Figure 1:**
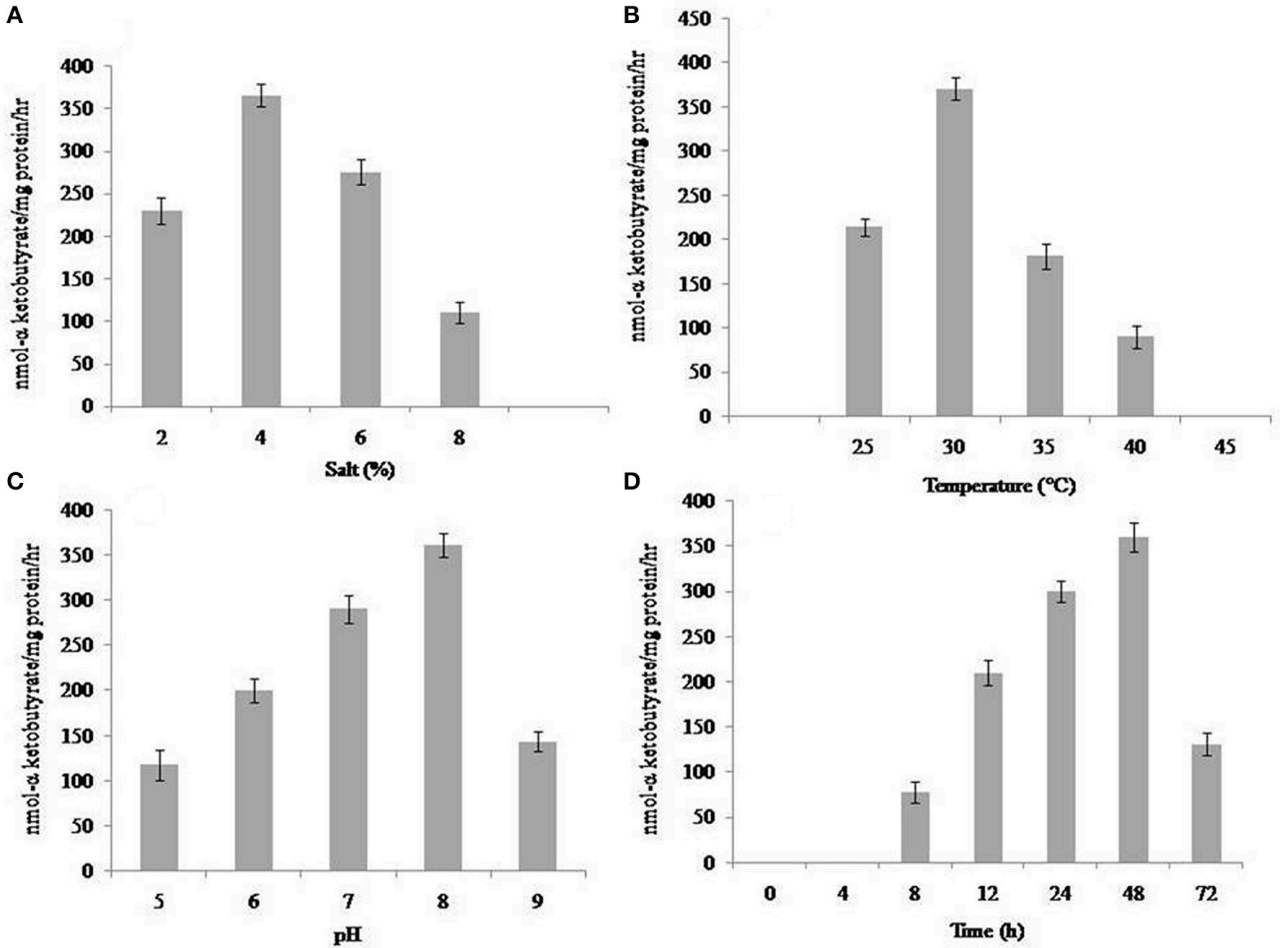
Evaluation of ACC deaminase activity of isolate SBP-9 in various physiological conditions; **(A)** salt stress **(B)** different temperatures **(C)** varying pH **(D)** incubation time. Data represent mean ± SD of triplicate sets.

The ACCD activity of the isolate SBP-9 was evaluated under various physiological conditions. Among different salt concentrations, highest ACCD activity of 365.38 ± 13.40 nmol α-KB mg^−1^ protein h^−1^ was observed in DF media supplemented with 4% of NaCl (Figure 1A). The increase in salinity from 2 to 4% increased the activity up to 59%, however, on further increase in NaCl concentration from 4 to 8%, activity was decreased up to 232%. Under varying temperature conditions, highest enzymatic activity was obtained at 30°C (370 ± 15 nmol α-KB mg^−1^ protein h^−1^), a further decrease in activity was recorded with rise in temperature (Figure 1B). Assessment of ACCD activity under various pH values demonstrated that pH 8.0 (361.37 ± 13.0 nmol α-KB mg^−1^ protein h^−1^) was optimum for enzymatic activity (Figure 1C). Higher enzymatic activity (360.90 ± 15.70 nmol α-KB mg^−1^ protein h^−1^) was recorded after 48 h of incubation (Figure 1D).

### Plant growth in response to bacterial inoculation

**Figure 2:**
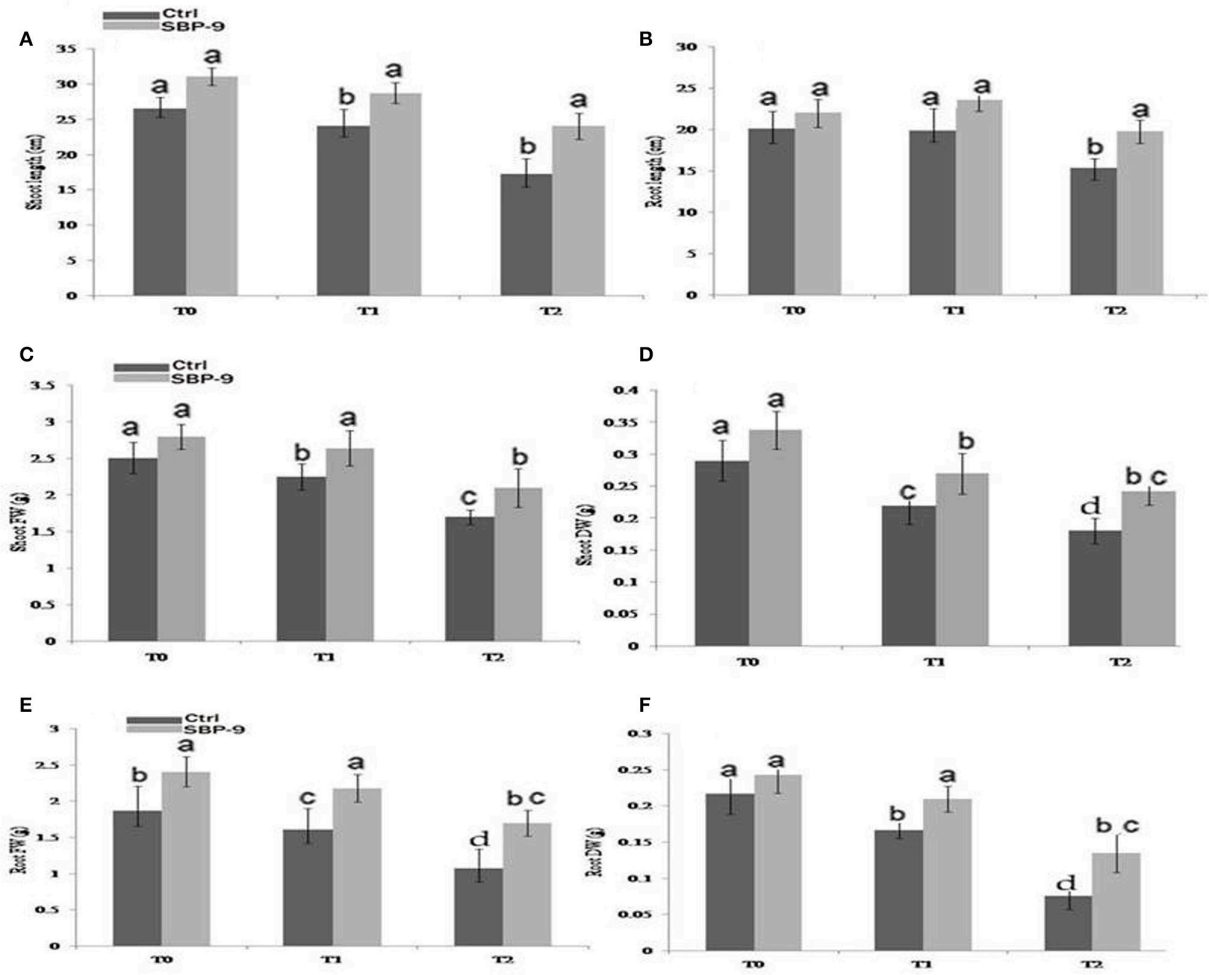
Effect of inoculation of isolate SBP-9 on plant growth and biomass content under different treatments T-0 (0 mM NaCl), T-1 (150 mM NaCl), T-2 (200 mM NaCl); **(A)** Shoot length **(B)** Root length **(C)** Shoot fresh weight **(D)** Shoot dry weight **(E)** Root fresh weight **(F)** Root dry weight. Each data represent the mean ± SD of triplicate sets of five measurements (*n* = 15). Different letters on the bar in each column represent the significant difference.

**Figure 3:**
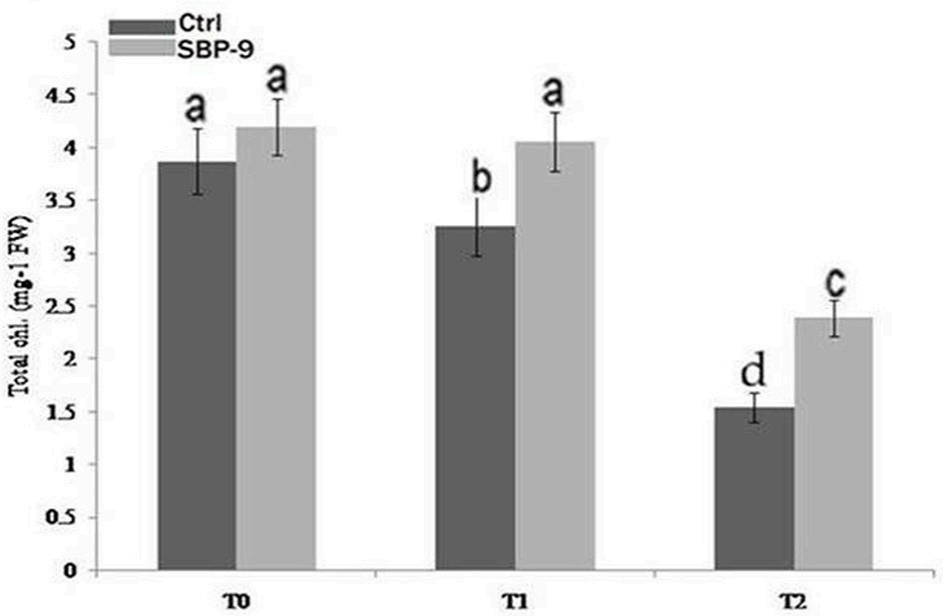
Effect of inoculation of isolate SBP-9 on total chlorophyll content under different treatments (T-0, T-1, T-2). Significant difference in each column has been shown by different letters.

Physiochemical characteristics of soil used for plant growth study have been summarized in Supplementary Table. 2. S*. maltophilia* SBP-9 enhanced both shoot and root growth of wheat plant under tested salinity stress. SBP-9 inoculation significantly improved the shoot length by 39% (*P* < 0.01), and 19.66% (*P* = 0.05) in T-2, and T-1 treatments as compared to corresponding control (Figure 2A). In response to SBP-9, root length was increased by 28.81% (*P* < 0.01) and 21% (*P* < 0.05) in T-2 and T-1 treatments as compared to respective control (Figure 2B). Bacterial application increased the biomass of wheat plant under both non-saline and saline stress conditions. Shoot fresh weight (SFW) increased by 18.40% (*p* = 0.05), and 24% (*P* < 0.05) at T-1 and T-2 treatments as compared to respective control (Figure 2C). Compared to corresponding control, SBP-9 inoculation increased the shoot dry weight (SDW) by 16.5% (*P* = 0.05), 23% (*P* < 0.05), and 34.4% (*P* < 0.01) at T-0, T-1, and T-2 treatments (Figure 2D). Similarly improvement in root fresh weight (RFW) was 29% (*P* < 0.01), 35.4% (*P* < 0.01), and 59% (*P* < 0.01) at T-0, T-1, and T-2 treatments, as compared to their respective control (Figure 2E). Following bacterial inoculation, root dry weight (RDW) increased by 31% (*P* < 0.01), and 70% (*P* < 0.01) at T-1, and T-2 treatments, as compared to respective control (Figure 2F). In response to SBP-9 inoculation, total chlorophyll content also increased at various treatments. It is evident from Figure 3 that highest increase in chlorophyll content of 55% (*P* < 0.01) was observed at treatment T-2, followed by 25% (*P* < 0.01) at treatment T-1, as compared to corresponding control.

### Ionic analysis in response to *S. maltophilia* SBP-9

**Figure 4:**
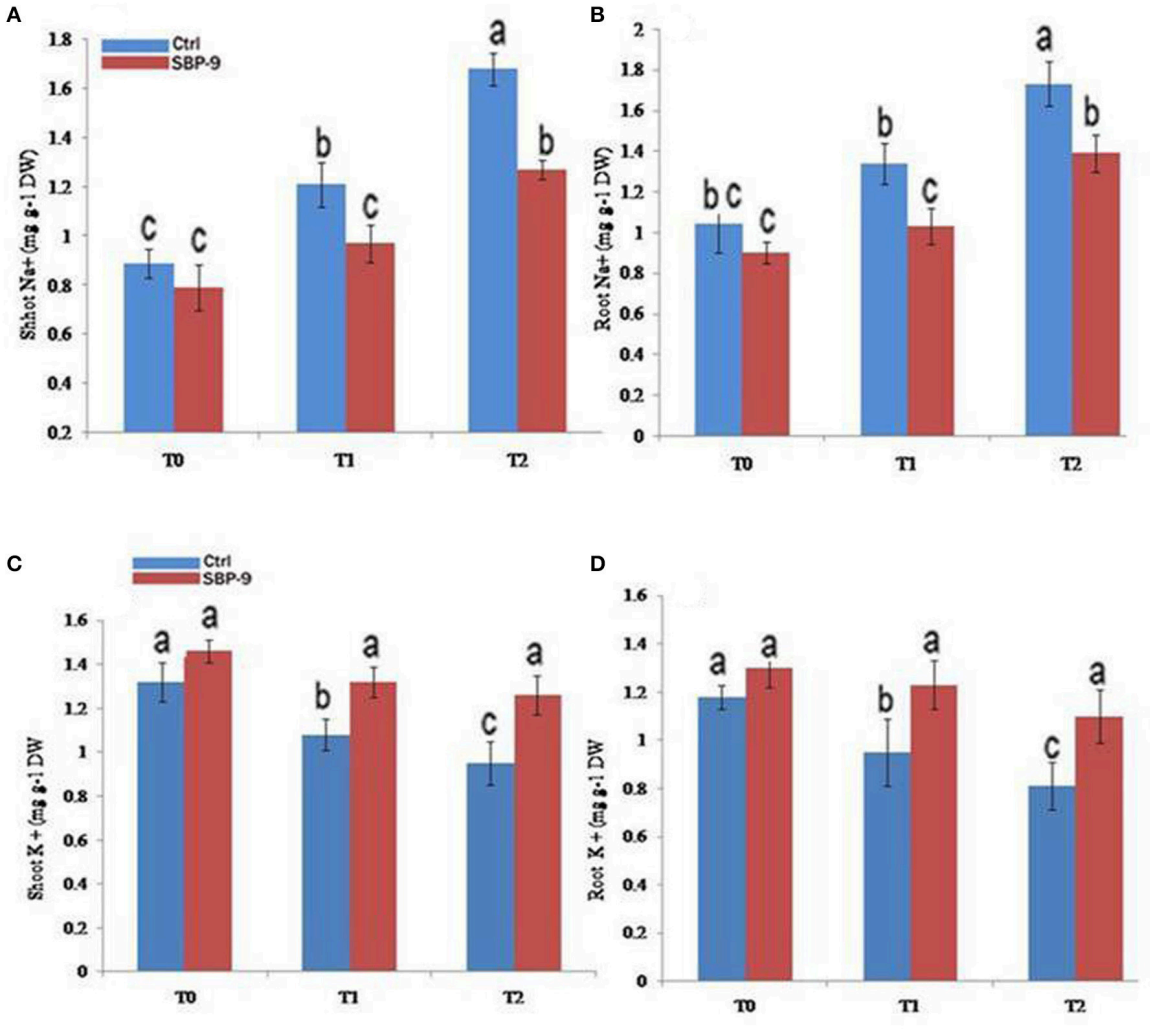
Effect of NaCl and inoculation with SBP-9 on ionic uptake by plants under different treatments T-0, T-1, T-2; **(A)** Shoot Na^+^ **(B)** Root Na^+^ **(C)** Shoot K^+^ **(D)** Root K^+^. Values are mean ± SD of triplicate sets of five measurements in triplicates (*n* = 15). Different letters on the bar in each column represent the significant difference.

Change in ionic contents particularly Na^+^ and K^+^ in response to SBP-9 inoculation was tested under non-saline and at a salinity level of 0 (T-0), 150 (T-1), and 200 mM (T-2) of NaCl. Bacterial inoculation did not significantly affect the shoot and root Na^+^ content under non-saline (T-0 treatment) condition. However, SBP-9 inoculation decreased the shoot Na^+^ content by 25% (*P* < 0.01), and 32.28% (*P* < 0.01), as well as root Na^+^ content by 30% (*P* < 0.01), and 24.5% (*P* < 0.05) in T-1 and T-2 treatments respectively, as compared to respective control (Figures 4A,B). Significant increase in shoot K^+^ content was 22% (*P* < 0.05) and 32.5% (*P* < 0.01) at T-1 and T-2 treatments, as compared to respective control. Furthermore, SBP-9 inoculation significantly increased the root K^+^ content by 29% (*P* < 0.01) and 35.8% (*P* < 0.01) at T-1 and T-2 treatments respectively, as compared to respective control (Figures 4C,D).

### Proline and MDA content

**Figure 5:**
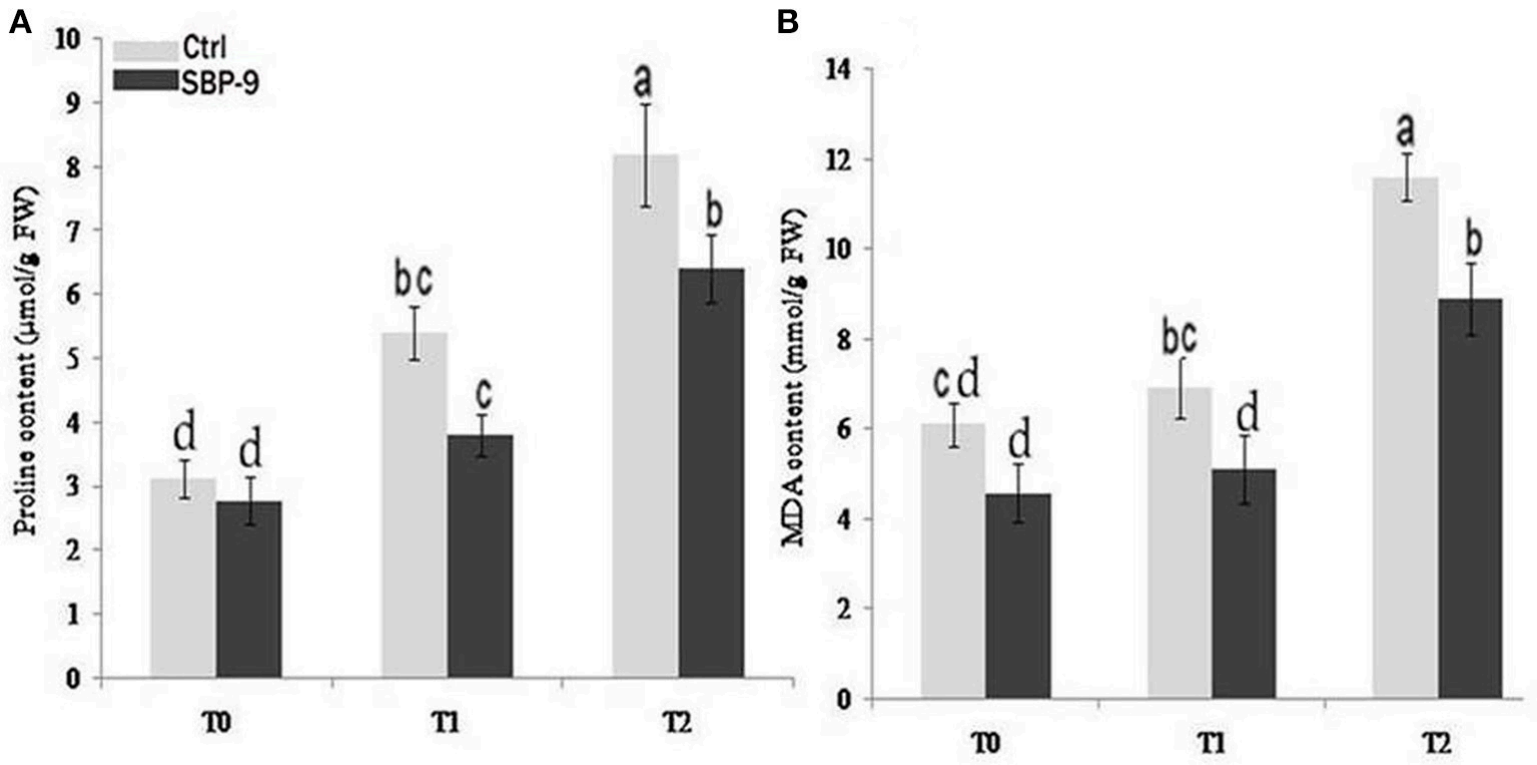
Effect of SBP-9 inoculation on proline **(A)** and malondialdehyde content **(B)** under different treatments; T-0 (0 mM NaCl), T-1 (150 mM NaCl), T-2 (200 mM NaCl). Values are mean ± SD of triplicate sets of five measurements in triplicate sets (*n* = 15). Different letters on the bar in each column represent the significant difference.

The observed results indicated that SBP-9 reduced the proline and MDA content under both non-saline and saline stress conditions. Proline content was decreased by 21.45% (*P* = 0.05) under non-saline condition (T-0 treatment) as compared to corresponding control. The highest decrease in proline content was 45.94% (*P* < 0.01) followed by 32.13% (*P* < 0.01) at T-1 and T-2 treatments as compared to corresponding control (Figure 5A).

Inoculation with SBP-9 significantly reduced the MDA content under both non-saline and salt stress conditions. MDA content was decreased by 24.39% (*P* < 0.05) under non-saline condition (T-0 treatment) as compared to control. Similarly, SBP-9 inoculation reduced the MDA content by 36% (*P* < 0.01), and 30% (*P* < 0.01) at T-1 and T-2 treatments respectively (Figure 5B).

### Antioxidative activities

**Figure 6:**
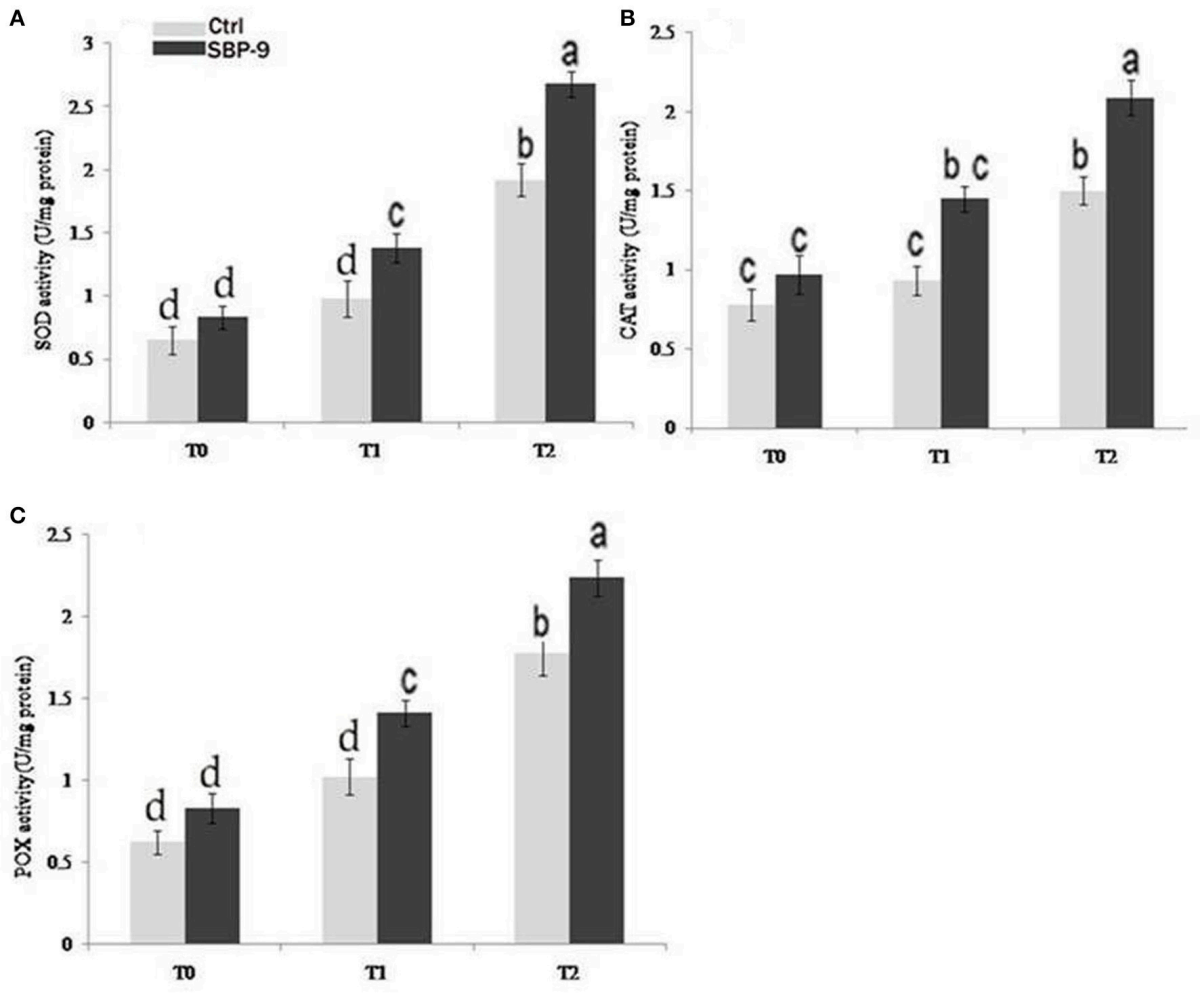
Effect of NaCl and SBP-9 inoculation on the antioxidant activities under tested treatments T-0, T-1, T-2; **(A)** Super-oxide dismutase (SOD) **(B)** Catalase (CAT) **(C)** Peroxidase (POX). Each value is mean of three replicates ± SD. Different letters on the bar in each column represent the significant difference.

A significant difference in the antioxidative enzyme activities of SBP-9-inoculated and control plants was observed under salinity stress conditions. Inoculation with SBP-9 significantly increased the antioxidative (SOD, CAT, POX) activities to alleviate the salinity induced free radical damages. SBP-9 inoculation slightly increased the SOD activity (27.70%) at T 0 treatment. However, the maximum increase in activity was 40.81% (*P* < 0.01) and 39.58% (*P* < 0.01) at T-1 and T-2 treatment as compared to respective control plants (Figure 6A). Considering the CAT enzyme, higher activity 55.91% (*P* < 0.01) was observed at treatment T-1, followed by 39.33% (*P* < 0.01) and 24.35% (*P* < 0.05) at treatments T-2 and T-0 respectively, as compared to corresponding control plants (Figure 6B). The highest significant (*p* = 0.05) increase in POX activities was 38.23% (*P* < 0.01) and 34% (*P* < 0.01) at T-1 and T-0 treatment as compared to respective un-inoculated plants. In T-2 treatment, bacterial inoculation significantly (*P* < 0.01) increased the POX activity of 25.84% (*P* < 0.05) as compared to respective control (Figure 6C).

### Induction of defense response

**Figure 7:**
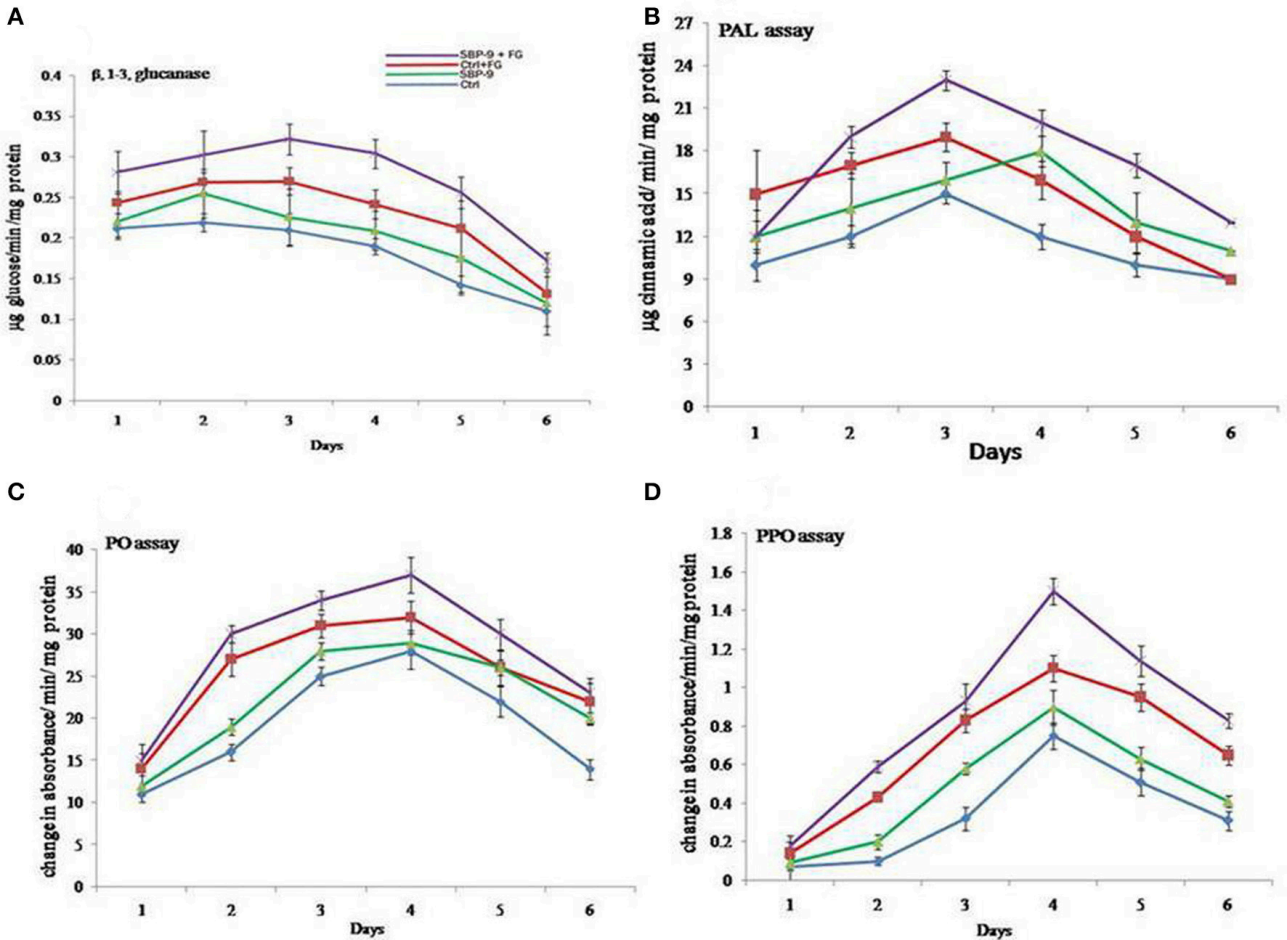
Augmentation of defense response in wheat by *S. maltophila* SBP-9 against *Fusarium graminearum* under controlled conditions; **(A)** β 1, 3-glucanase assay **(B)** Phenylalanine ammonia lyase assay **(C)** Peroxidase assay **(D)** Polyphenol oxidase assay.

In the plant growth experiment, the bacterial isolate significantly increased the defense enzymes in wheat plant challenged with the pathogen. Plants pre-treated with SBP-9 and challenge-inoculated with fungal pathogen showed a concurrent increase in β-1, 3 glucanase, PAL, PO, and PPO. Upon pathogen challenge in bacterized wheat plants, β-1, 3 glucanase activity started to increase up to 3rd day (332 ± 20 ng glucose/min/mg protein), thereafter declined gradually (Figure 7A). Similarly, wheat plant inoculated with pathogen in bacterium-primed plants also showed increase in the PAL activity. The higher induced activity was observed on 3rd day (23 ± 2.1 nmol of trans-cinnamic acid) (Figure 7B). Wheat plants treated with the isolate SBP-9 alone also had higher PAL activity, however the activity level was less during the first 3 days as compared to control plants challenged with a fungal pathogen. PO activity also increased in bacteria-treated plants challenged with the pathogen. The maximum activity was observed at 4th day after pathogen inoculation (Figure 7C). A similar pattern of increased PPO activity was recorded in bacterized wheat plants challenged with the pathogen. The plants of uninoculated control showed the lowest enzyme activity among all treatments (Figure 7D).

### Colonization

Colonization efficiency of the bacterium was determined by plate counting after 15 days of plant growth. The associated bacterium was found in a range of 1.8 × 10^3^ CFU g^−1^ of the root. No bacterial colonies were recovered from uninoculated control plants. In addition, for the ERIC-PCR profile obtained from total DNA of treated plants was identical to that of a pure culture of *S. maltophilia* SBP-9, which indicated that the bacterium had successfully colonized the plants (Supplementary Figure 4).

## Discussion

The present work is an attempt to evaluate the biotic and abiotic stress tolerance conferred by ACC-deaminase bacterium *S. maltophilia* SBP-9 in wheat plants. To the best of our knowledge, the present study is the first to report *Stenotrophomonas* sp. having ACC deaminase activity and conferring induced systemic tolerance in wheat plant under salinity stressors. Plant rhizosphere is a preferred niche for soil microorganisms which is relatively rich in organic substrates, for stimulating microorganism growth and attracts diverse genera of microbial species. In response to saline stress, ACC is transported by the xylem to shoots where it is oxidized to ethylene Jackson, 1985). Meantime ACC is secreted by roots, which stimulates the proliferation inoculated ACCD producing bacteria (Grichko and Glick, 2001; Dobbelaere et al., 2003). From the previous finding, it was observed that exogenous application of ethylene or its precursor ACC decreased the root growth (Nukui et al., 2000), however bacteria equipped with ACC deaminase minimize the level of “stress ethylene” and thus confer resistance to various biotic and abiotic stresses. According to previous report (Glick et al., 1994) *AcdS*^−^ mutant (lacking ACC deaminase activity) of PGPR strain *Pseudomonas putida* GR12-2 lost the ability to promote canola root elongation. Similarly, Sergeeva et al. (2006) proposed that ACC deaminase activity has a dual role in growth promotion and tolerance to high salt stress in transgenic canola plants.

Following above evidences, it is assumed that plant growth promotion by *S. maltophilia* SBP-9 in wheat seedlings exposed to salinity stress might be attributed to the ACC-deaminase activity, which in turn reduces the synthesis of ethylene. Isolate SBP-9 showed quite high ACCD activity, having >20 nmol of α-KB mg^−1^ h^−1^ which is enough to trigger systemic tolerance under stress conditions (Penrose and Glick, 2003). The strain was found to produce phytohormone IAA and solubilize phosphate that supports the plant growth stimulation under adverse condition like salt (Egamberdieva, 2009; Ramadoss et al., 2013). Imran et al. (2014) reported that bacterial isolates having multiple beneficial traits is better than having the single trait. Many of the rhizobacteria are known to release iron-chelating siderophores into the rhizosphere of its/their host plant that influence the uptake and availability of other metal like Zinc (Zn), Copper (Cu), and Iron (Fe) (Grichko et al., 2000; Egamberdiyeva, 2007; Dimkpa et al., 2009).

The ACCD activity of SBP-9 was characterized under different physiological conditions. Among different salt and temperature conditions, higher activity was recorded at 4% salinity and 30°C temperature. Significant plant growth promotion was achieved under salt stress on inoculation of isolate SBP-9 which could result from one or more growth promoting properties of the inoculated bacterium. Cheng et al. (2007) suggested that inoculation with ACCD bacterium *P. putida* UW4 enhances the various physiological parameters of *Brassica napus* under inhibitory level of salinity stressors. Likewise, significant increase in plant growth and number of leaves of *Limonium sinense* was also observed following inoculation of ACCD bacteria under salt stress (Sheng et al., 2014). In our study, SBP-9 was found as an efficient promoter of wheat plant especially under salt exposure (150 and 200 mM NaCl). In addition, salt stress also hampers the photosynthetic mechanism due to chlorophyll peroxidation (Tuna et al., 2008; Barry, 2009). However, inoculation of isolate SBP-9 significantly improved the leaf chlorophyll content as compared to uninoculated control under both non-saline and salinity conditions, illustrating the ability of the strain to counteract the salinity stressors. Our results are in concurrence with the previous study where ACCD producing bacteria stimulate the plant growth under varying salinity stressors (Barnawal et al., 2014). However, Contesto et al. (2008) suggested that *Arabidopsis* plants inoculated with ACCD mutant and its wild type counterpart did not show any difference in plant growth promoting effects under stress conditions. Thus, future work is required to establish the role of ACC deaminase in isolate SBP-9 by raising ACCD mutant of isolate SBP-9 and its effect on plant growth.

Exclusion of Na^+^ and influx of K^+^ are the plant's strategies for mitigating the salinity induced oxidative stress (Shabala and Cuin, 2008). In our study, inoculation with isolate SBP-9 significantly decreased the accumulation of Na^+^ and increased K^+^/Na^+^ levels in both shoots and roots of the wheat plant under salinity stress. Our result is in congruence to a previous study where inoculation of ACCD bacteria significantly improved the K^+^ content in tomato plants under salt stress (Mayak et al., 2004). Previous study of Zhang et al. (2008) suggested that *Arabidopsis* inoculation with *Bacillus subtilis* GB03 decreased the Na^+^ content (54%) by down-regulating HKT1 expression in roots and up-regulating HKT1 expression in shoots to enhance shoot-to-root Na^+^ recirculation. Therefore, correlating these changes in response to ACCD producing bacteria could provide the evidence of plant growth regulation in physiologically diverse conditions (Rodriguez-Rosales et al., 2008).

Alleviation of salinity-induced oxidative damages with the use of antioxidant enzymes is an important strategy of plants for increasing its tolerance to stress conditions. In the present work, increased activities of various antioxidant enzymes following bacterial inoculation illustrated that these enzymes play a crucial role in protection of plants under salinity like stresses. *A. xylosoxidans* increases the antioxidant activity in *Catharanthus roseus* (Karthikeyan et al., 2008) and in the *Solanum melongena* inoculated with *Pseudomonas* sp. DW1 (Fu et al., 2010). The bacterial SOD facilitates the removal of free radicals and plays an important role in their survival in the rhizosphere (Wang et al., 2007). The major breakdown product of SOD is H_2_O_2_, which is a toxic lipid peroxidant, but can be eliminated by activities of CAT and POX antioxidant enzymes. The POX activity plays a major role in eliminating the stress induced H_2_O_2_ and malondialdehyde level, thus protecting the cell membrane integrity. Our data showed that activities of SOD, CAT, and POX enzymes in leaves of SBP-9 inoculated plants were higher compared to uninoculated plants under salinity stress. The increase in enzyme activities was probably due to the fact that bacterial inoculation stimulated the synthesis of these enzymes (Wang et al., 2010).

The accumulation of proline in response to salt stress protects the cell membrane, stabilizes the structure of the protein and scavenges the free hydroxyl radicals (Claussen, 2005). In the present study, the SBP-9-inoculated plants showed lower proline levels as compared to uninoculated control plants. This decrease in proline level in SBP-9-inoculated plants indicated that ACC deaminase producing bacterial-inoculated plants were less affected by salinity. Soil salinity increases the generation of ROS in plants which enhance the membrane lipid peroxidation and increase the MDA content (Koca et al., 2006; Yazici et al., 2007). Therefore, leaf MDA content is usually used to evaluate plant tolerance to salinity (Luna et al., 2000). The decrease in MDA content in SBP-9 inoculated plants indicates that bacterial inoculation protects the plants from the imposed salt stress.

Beneficial bacterial-mediated induced systemic resistance is associated with induction of various defense enzymes like β 1-3-glucanase, PAL, PPO, and PO (Meena et al., 2000). The previous investigation of Umamaheswari et al. (2010) showed the increased accumulation of these defense enzymes in watermelon plants confronted with *Alternaria alternate* in the presence of biocontrol microorganisms. However, in most of the study very little is known about the mechanisms governing the ISR response. The production of various defense enzymes in the presence of SBP-9 might illustrate its role in the generation of resistance to pathogen infection. The increased level of β 1-3-glucanase, PAL, PPO, and PO might play a key role in pathogen suppression in bacterium-primed plants challenged with the pathogen. The defense enzymes PAL and PO are involved in the biosynthesis of phytoalexins and phenolics that are primarily responsible for disease resistance (Daayf et al., 1997). PAL favors the formation of trans-cinnamic acid from phenylalanine, an intermediate in salicylic acid biosynthesis (Ryals et al., 1996). The activities of PO and PPO are linked to the generation of hydrogen peroxide and lignification during infection which inhibits phytopathogens directly or induce the generation of free radicals that inhibit the proliferation of pathogens (Silva et al., 2004). Besides, PPO oxidizes phenolics to toxic quinines and is involved in the terminal oxidation of diseased plant tissue (Kosuge, 1969).

Additionally the tested isolate SBP-9 showed all forms of motility which are required for the chemotactic responses and colonization (VandeBroek et al., 1998; Lugtenberg and Kamilova, 2009). Colonization ability of the isolate can provide the maximum benefits to plants as it establishes a close relationship with the plant host than rhizosphere and modulates the defense response to cope with adverse conditions. Effective colonizing bacterium encounters a protective environment where they could have a better survival and therefore more prolonged activity (Hardoim et al., 2008).

## Conclusion

The observed results of present study demonstrates that use of the inoculation with multifarious plant growth promoting bacterium *S. maltophilia* significantly improves the growth, ionic balance and biochemical parameters of plants, thus allowing them to cope with imposed salinity stress. The effect of beneficial rhizobacteria on plant growth improvement are well-known, however the physiological and molecular mechanisms still need to be explored for better utilization of these microorganisms. The association of these microorganisms to plants at the genetic level will provide valuable insight for microbial mediated enhanced salinity tolerance and this may help to pave the way for the commercial application of the microorganism at field level. The selected strain in the present study should be tested for its capability to enhance plant growth in unsterilized soil and at field level also. Furthermore, the efficacy of strain can be tested on the other plants besides of wheat. Nevertheless, mechanistic studies between PGPR and plant are still in need to investigate the ways by which PGPR exert beneficial effects on plants.

## Author contributions

RS performed the experiments and PJ mentored the research work.

### Conflict of interest statement

The authors declare that the research was conducted in the absence of any commercial or financial relationships that could be construed as a potential conflict of interest.

## Supplementary material

Supplementary Figure 1 Phylogenetic tree showing the relationship of *S. maltophilia* SBP-9 to closely related bacteria. PCR amplified amplicon of partial 16S rRNA gene of SBP-9 was sequenced and used for the construction of a phylogenetic tree. The 16S rRNA gene sequence of closely related species was obtained from NCBI GenBank database. The tree was obtained using neighbor- joining method of software packages Mega version 6.0, at the bootstrap value of (*n* = 1,000).

Supplementary Figure 2 Siderophore production by the test isolate *S. maltophila* SBP-9 on CAS-agar plate.

Supplementary Figure 3 *nif*-H gene amplification in *S. maltophila* SBP-9.

Supplementary Figure 4 Tracking of colonization of *S. maltophila* SBP-9 through ERIC-PCR profile of bacterium colonized on wheat plants and confirmation of its identity using profile of pure culture (Lane M: DNA ladder SM0311, Lane A: control DNA, Lane B: DNA of *S. maltophila* SBP-9 isolated from treated plant).
