# Supplementary figures and images for: The PGPR Stenotrophomonas maltophilia SBP-9 Augments Resistance against Biotic and Abiotic Stress in Wheat Plants
Source: Front Microbiol. 2017 Oct 9;8:1945. doi: 10.3389/fmicb.2017.01945 (PMC5640710; doi:10.3389/fmicb.2017.01945)

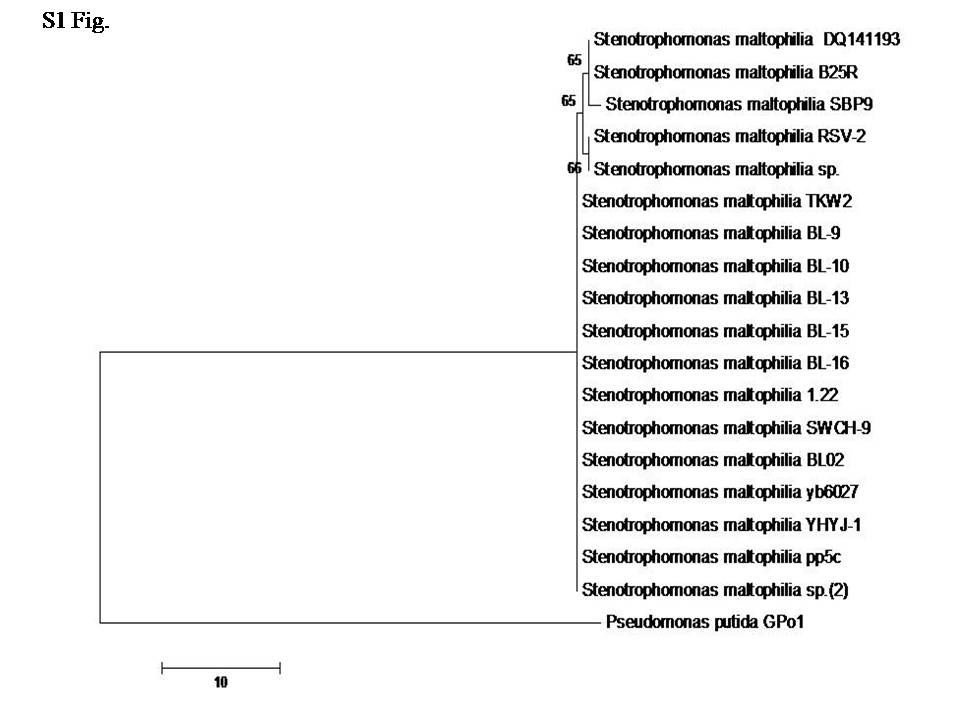

Supplement: Supplementary Figure 1 — Phylogenetic tree showing the relationship of S. maltophilia SBP-9 to closely related bacteria. PCR amplified amplicon of partial 16S rRNA gene of SBP-9 was sequenced and used for the construction of a phylogenetic tree. The 16S rRNA gene sequence of closely related species was obtained from NCBI GenBank database. The tree was obtained using neighbor- joining method of software packages Mega version 6.0, at the bootstrap value of (n = 1,000). [file Image1.JPEG]

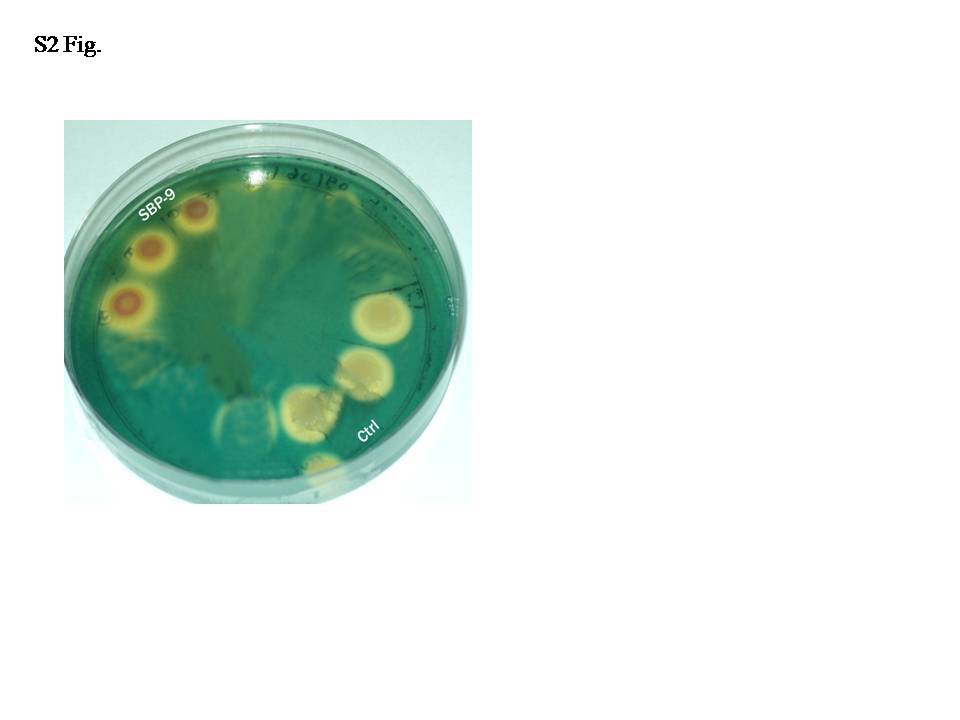

Supplement: Supplementary Figure 2 — Siderophore production by the test isolate S. maltophila SBP-9 on CAS-agar plate. [file Image2.JPEG]

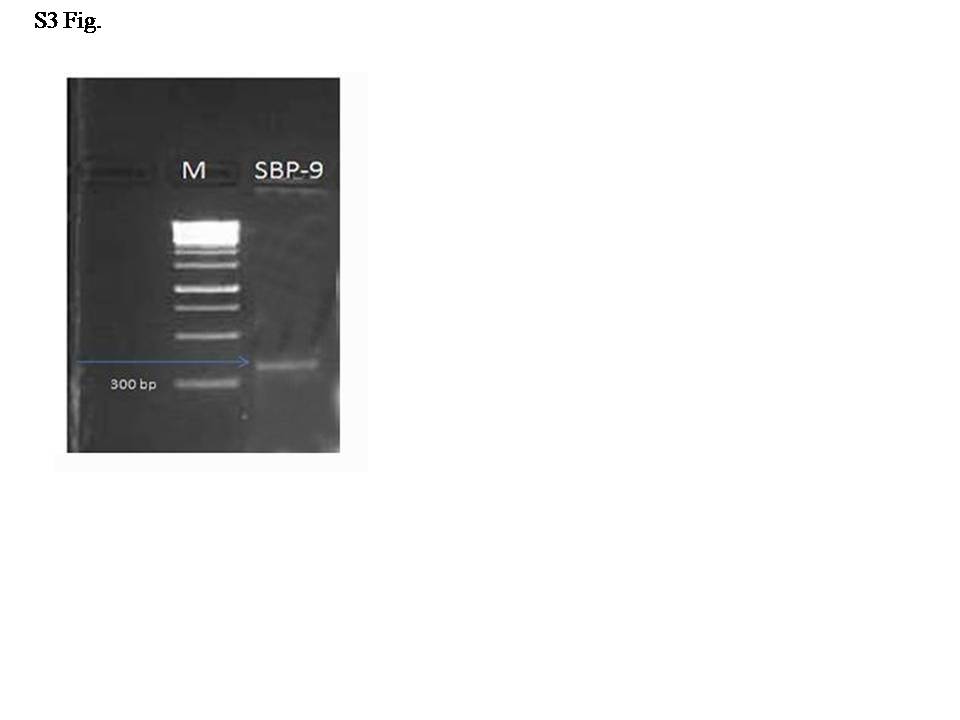

Supplement: Supplementary Figure 3 — nif-H gene amplification in S. maltophila SBP-9. [file Image3.JPEG]

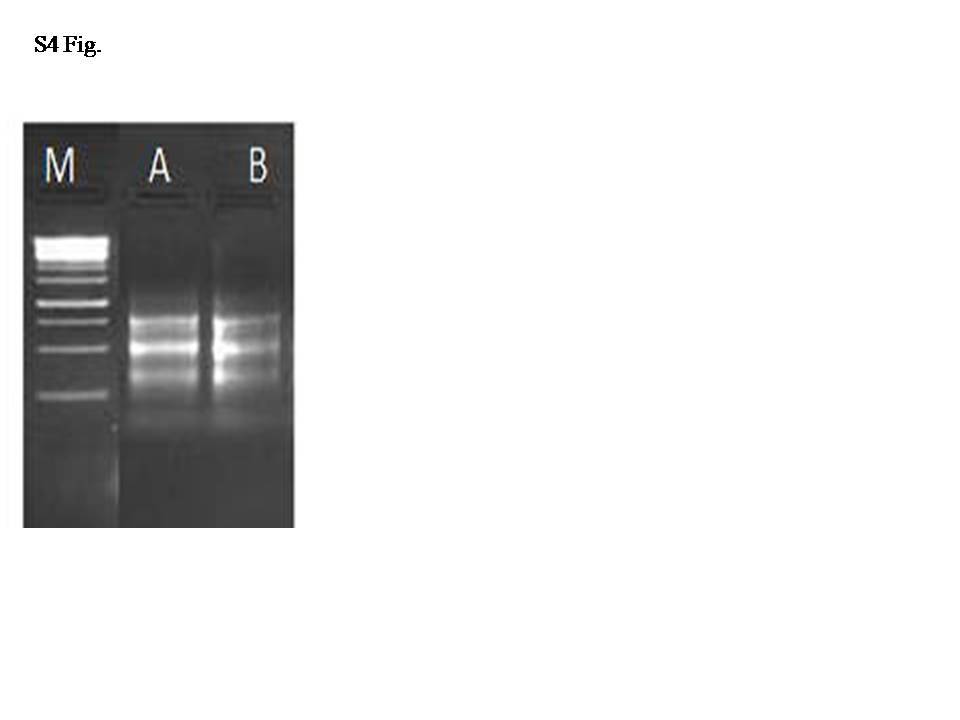

Supplement: Supplementary Figure 4 — Tracking of colonization of S. maltophila SBP-9 through ERIC-PCR profile of bacterium colonized on wheat plants and confirmation of its identity using profile of pure culture (Lane M: DNA ladder SM0311, Lane A: control DNA, Lane B: DNA of S. maltophila SBP-9 isolated from treated plant). [file Image4.JPEG]
