# Supplementary material for: The PGPR Stenotrophomonas maltophilia SBP-9 Augments Resistance against Biotic and Abiotic Stress in Wheat Plants
Source: Front Microbiol. 2017 Oct 9;8:1945. doi: 10.3389/fmicb.2017.01945 (PMC5640710; doi:10.3389/fmicb.2017.01945)
Supplement: Supplementary file 5 [file Table1.docx]

**Supplementary Table 1. Carbohydrate utilization behaviour of isolate SBP-9**

**Carbohydrate Activity Carbohydrate Activity**

Lactose + α-Methyl-D-mannoside -

Xylose + Xylitol -

Maltose + ONPG +

Fructose + Esculin hydrolysis +

Dextrose + D-Arabinose +

Galactose + Citrate utilization -

Raffinose + Malonate utilization -

Trehalose + Sorbose -

Melibiose + Inulin +

Sucrose + Mannose +

L-Arabinose + Arabitol +

Sodium gluconate - Erythritol -

Glycerol + α-Methyl-D-glucoside -

Salicin + Rhamnose +

Dulcitol - Cellobiose +

Inositol - Melezitose **-**

Sorbitol + Adonitol +

Mannitol +

+, positive; -, negative
