# Supplementary material for: The PGPR Stenotrophomonas maltophilia SBP-9 Augments Resistance against Biotic and Abiotic Stress in Wheat Plants
Source: Front Microbiol. 2017 Oct 9;8:1945. doi: 10.3389/fmicb.2017.01945 (PMC5640710; doi:10.3389/fmicb.2017.01945)
Supplement: Supplementary file 6 [file Table2.docx]

**Supplementary Table 2. Physiochemical properties of soil used for pot study**

**Parameter Value**

pH 7.10±0.03

EC 0.168±0.01ds m^-1^

Olsen P 34.9 ±1.7mg kg^-1^

Total N 60 ±1.8 mg kg^-1^

Total K 121.0 ±3.6 mg kg^-1^

Zn 0.209 ±0.002 mg kg^-1^

Cu 0.127 ±0.001mg kg^-1^

Fe 2.81 ±0.01 mg kg^-1^

Mn 0.950 ±0.03 mg kg^-1^
